# Anti-SARS-CoV-2 IgA and IgG in human milk after vaccination is dependent on vaccine type and previous SARS-CoV-2 exposure: a longitudinal study

**DOI:** 10.1186/s13073-022-01043-9

**Published:** 2022-04-21

**Authors:** Marta Selma-Royo, Christine Bäuerl, Desirée Mena-Tudela, Laia Aguilar-Camprubí, Francisco J. Pérez-Cano, Anna Parra-Llorca, Carles Lerin, Cecilia Martínez-Costa, Maria Carmen Collado

**Affiliations:** 1 Department of Biotechnology, Institute of Agrochemistry and Food Technology-National Research Council (IATA-CSIC), 46980 Paterna, Valencia Spain; 2 Department of Nursing, Nursing Research Group, Universitat Jaume I, Castellón, Spain; 3 LactApp Women Health, Barcelona, Spain; 4 Physiology Section, Department of Biochemistry and Physiology, Faculty of Pharmacy and Food Science and Institute of Research in Nutrition and Food Safety (INSA), University of Barcelona (UB), 08028 Barcelona, Spain; 5 Health Research Institute La Fe, Neonatal Research Group, Spain and University and Polytechnic Hospital La Fe, Division of Neonatology, 46026 Valencia, Spain; 6 Endocrinology Department, Institut de Recerca Sant Joan de Déu, Hospital Sant Joan de Déu, 08950 Barcelona, Spain; 7 Department of Pediatrics, Hospital Clínico Universitario, University of Valencia, Valencia, Spain; 8 Nutrition Research Group of INCLIVA, 46010 Valencia, Spain

**Keywords:** Breast milk, SARS-CoV-2, Antibodies, Immunoglobulins, Vaccines

## Abstract

**Background:**

Breast milk is a vehicle to transfer protective antibodies from the lactating mother to the neonate. After SARS-CoV-2 infection, virus-specific IgA and IgG have been identified in breast milk, however, there are limited data on the impact of different COVID-19 vaccine types in lactating women. This study is aimed to evaluate the time course of induction of SARS-CoV-2-specific IgA and IgG in breast milk after vaccination.

**Methods:**

In this prospective observational study in Spain, 86 lactating women from priority groups receiving the vaccination against SARS-CoV-2 were included. Breast milk samples were collected longitudinally at seven or eight-time points (depending on vaccine type). A group with confirmed SARS-CoV-2 infection (*n*=19) and a group of women from pre-pandemic time (*n*=20) were included for comparison.

**Results:**

Eighty-six vaccinated lactating women [mean age, 34.6 ± 3.7 years] of whom 96% were Caucasian and 92% were healthcare workers. A total number of 582 milk samples were included, and vaccine distribution was BioNTech/Pfizer (BNT162b2, *n*=34), Moderna (mRNA-1273, *n*=20), and AstraZeneca (ChAdOx1 nCoV-19, *n*=32). For each vaccine, 7 and 8 longitudinal time points were collected from baseline up to 30 days after the second dose for mRNA vaccines and adenovirus-vectored vaccines, respectively. A strong reactivity was observed for IgG and IgA after vaccination mainly after the 2^nd^ dose. The presence and persistence of specific SARS-CoV-2 antibodies in breast milk were dependent on the vaccine type, with higher IgG and IgA levels in mRNA-based vaccines when compared to AstraZeneca, and on previous virus exposure. High intra- and inter-variability were observed, being relevant for IgA antibodies. In milk from vaccinated women, anti-SARS-CoV-2 IgG was significantly higher while IgA levels were lower than in milk from COVID-19-infected women. Women with previous COVID-19 increased their IgG antibodies levels after the first dose to a similar level observed in vaccinated women after the second dose.

**Conclusions:**

COVID-19 vaccination induced anti-SARS-CoV-2 IgA and IgG in breast milk with higher levels after the 2^nd^ dose. Levels of anti-SARS-CoV-2 IgA and IgG are dependent on the vaccine type. Further studies are warranted to demonstrate the protective antibody effect against COVID-19 in infants from vaccinated and infected mothers.

**Trial registration:**

NCT04751734 (date of registration is on February 12, 2021)

**Supplementary Information:**

The online version contains supplementary material available at 10.1186/s13073-022-01043-9.

## Background

Breastfeeding is the most important postnatal link between mothers and infants, being the best source of nutrition with effects on infant health and development [1] including the maturation of the neonatal immune system, which is especially relevant in the context of coronavirus disease 2019 (COVID-19) vaccination. Similar to other infective processes [2], some studies have reported the presence of specific antibodies in milk after SARS-CoV-2 infection. In particular, a rapid and strong antibody response is induced after maternal SARS-CoV-2 infection, with the subsequent accumulation of substantial amounts of specific neutralizing secretory IgA (sIgA) and other antibody types in breast milk [3–5].

Europe initiated the vaccination program against COVID-19 on December 27^th^, 2020 [6]. In Spain, health care workers were priority groups to receive the vaccines, including breastfeeding women. The first available vaccines were mRNA-based vaccines BNT162b2 mRNA and mRNA-1273; later on, an adenovirus-vectored vaccine (ChAdOx1 nCoV-19) became available [7]. While these vaccines protect against severe COVID-19 disease in adult populations [8–10], limited data are available on lactating women as they were not included in vaccine trials [1]. Despite this lack of information, main organizations including the Centers for Disease Control and Prevention have recommended lactating women to be immunized [11, 12]. Preliminary studies showed that mRNA-based vaccines induced anti-SARS-CoV-2 antibodies in breast milk [12–14]. However, several questions remain still open including the extent of the vaccination effect, whether there is a differential response depending on the vaccine type and the impact of vaccination on women with past SARS-CoV-2 infection.

We investigated whether maternal immunization with the available vaccines, mRNA-based vaccines BNT162b2 mRNA and mRNA-1273 and also the adenovirus-vectored vaccine (ChAdOx1 nCoV-19), resulted in the secretion of anti-SARS-CoV-2-specific IgA and IgG into breast milk and also evaluated any potential adverse events among women and their infants. Furthermore, we compared milk antibody levels from vaccinated women to those found in naturally immunized women after COVID-19.

## Methods

### Study population and design

This is a prospective observational, longitudinal, and multicenter study in lactating women receiving vaccination against SARS-CoV-2 infection in Spain (ClinicalTrials.gov Identifier: NCT04751734; URL: https://clinicaltrials.gov/ct2/show/NCT04751734). The primary outcome measure of the study was the presence and levels of anti-SARS-CoV-2 antibodies in breast milk from vaccinated women.

The recruitment period started in January 2021 and it is still ongoing. Participants were breastfeeding women within the vaccination priority groups (frontline health care workers) established by the Spanish Government. Participants were breastfeeding women recruited at hospitals and health care centers as well as by using specific tools such as LactApp (an app dedicated to breastfeeding and motherhood) [15] and social media (i.e., Twitter and MilkCORONA study members’ webpages). Women were excluded if the cessation of breastfeeding and/or vaccine side-effects required specific treatment and/or hospitalization. Participants received information and written consent was obtained before enrollment. All procedures conformed to the principles of the Helsinki Declaration, and they were performed in accordance with the ethical standards approved by the Ethical Committee of the Hospital Clínico Universitario (Ref. 2020/133), the Hospital Sant Joan de Déu (Ref. PIC-94-21), and the Spanish National Research Council-CSIC (Ref. 061/2021).

Participants received two doses of mRNA vaccines (BNT162b2 mRNA, BioNTech/Pfizer and mRNA-1273, Moderna) or of adenovirus-vectored vaccine (ChAdOx1 nCoV-19, Oxford/AstraZeneca). Human milk samples were collected longitudinally at seven-time points depending on the vaccines: pre-vaccination (T1-0): 0 weeks, 1 week (T1-1), 2 weeks (T1-2), and 3–4 weeks (T1-3) post the 1^st^ dose of vaccine; 1 week (T2-1), 2 weeks (T2-2), and 3–4 weeks (T2-3) post 2^nd^ dose. The time course of BioNtech/Pfizer and Moderna vaccines were 21 and 28 days, respectively. For the adenovirus-vectored vaccine, samples were also collected before administration of the 2^nd^ dose (T2-0) due to a longer interval of 3 months between doses. Furthermore, a group with confirmed SARS-CoV-2 infection (*n*=19) (ClinicalTrials.gov Identifier: NCT04768244) from a previous [16] study was included. The time elapsed between collection of samples and PCR-diagnosis (*n*=17) of SARS-CoV-2 was 22 days (median, 25th and 75th percentile 3.5–22.0 days, respectively), and in two mothers, SARS-CoV-2 infection was diagnosed by serological test and samples collected on days 17 and 274 post-diagnosis. Furthermore, a control group of women not exposed to SARS-CoV-2 from pre-pandemic time [17] (*n*=20) (ClinicalTrials.gov Identifier: NCT03552939) was also included.

Maternal, pregnancy, and birth characteristics were collected as covariates for descriptive purposes and matched on maternal characteristics for associations between SARS-CoV-2 positive women and pre-pandemic groups and neonatal outcomes (Table 1).

**Table 1:** Characteristics of the participants included in the study

	BioNtech/Pfizer *N*=34	Moderna *N*=20	Oxford/AstraZeneca *N*=32	*p*-value
Maternal characteristics				
Age (years)	34.2 ±4.1	34.8 ±3.1	34.7 ±3.6	0.802
Chronic diseases (hypothyroidism, heart disease)	8 (23.5%)	5 (33.3%)	6 (18.75%)	0.841
Medication	5 (14.7%)	3 (14.3%)	3 (9.4%)	0.782
Gestational age (weeks)	39.2 ±2.1	39.4 ±1.4	39.9 ±1.4	0.246
Education degree (high-degree university studies)	32 (94.1%)	19 (95.2%)	29 (90.6%)	0.792
SARS-CoV-2-positive PCR before vaccine	6 (17.1%)	2 (9.5%)	0 (0.0%)	-
Infant characteristics				
Gender (female, %)	10 (29.4%)	9 (45%)	17 (53.1%)	0.141
Mode of birth (C-section, %)	10 (29.4%)	6 (30%)	6 (18.7%)	0.535
Weight at birth (g)	3308.4 ±420.6	3495.0 ±616.8	3457.8 ±369.1	0.262
Length at birth (cm)	50.8 ±2.1	50.5 ±2.4	51.1 ±1.8	0.591
Infant age at maternal vaccination (month)	14.3±11	11.0±5.2	12.4±7.6	0.385
Exclusive breastfeeding at maternal vaccination (%)	5 (14.7%)	3 (15%)	4 (12.5%)	0.956

Categorical data is presented as positive cases (% of the total population). Chi-squared test and one-way ANOVA were used to test significant differences in clinical categorical and continuous variables, respectively, according to vaccine

### Human milk collection and processing

Breast milk collection was performed by each woman at home following the recommended procedures [18]. Then, milk was collected mainly by the use of a sterile pumper in sterile bottles to normalize the collection among participants and morning collection was recommendable. Breast milk samples were stored immediately at −20 °C and later stored at −80 °C as detailed elsewhere [16]. In brief, samples were thawed and centrifuged, the whey milk was aliquoted and frozen at −80 °C until use. COVID-19 and pre-pandemic milk samples were processed in the same manner.

### Detection of specific SARS-CoV-2 antibodies in breast milk

Antibodies against the receptor-binding domain (RBD) SARS-CoV-2 Spike protein were determined as previously described [16]. Briefly, 96-well ELISA immunoplates (Costar) were coated with RBD protein (2 μg/mL) and incubated at 4 °C overnight. RBD protein was produced under HHSN272201400008C and obtained through BEI Resources, NIAID, NIH: Spike Glycoprotein Receptor Binding Domain (RBD) from SARS-related coronavirus 2, Wuhan-Hu-1 with C-Terminal Histidine Tag, recombinant from HEK293T Cells, NR-52946. Coated plates were blocked in 3 % (w/v) milk powder in phosphate-buffered saline (PBS) containing 0.1 % Tween 20 (PBS-T) for 3 h. Then, diluted samples in 1 % (w/v) milk powder in PBS-T were added, incubated for 2 h at room temperature, and washed with PBS-T before the addition of horseradish peroxidase-conjugated secondary antibodies. For detection, anti-human IgA (α-chain-specific) HRP antibody (Thermo-Fisher Scientific; A18781; 1:6.000) and anti-human IgG (Fc specific) HRP antibody (Sigma-Aldrich; A0170; 1:4.000) were used. Bound antigen-specific antibodies were detected with 100 μL 3,3′,5,5′-tetramethylbenzidine, and reactions were stopped with 50 μL of 2M sulfuric acid. Absorbance at 450 nm was read in a ClarioStar (BMG Labtech) microplate reader using the path length correction mode. Standards for interpolation of arbitrary units (AU) were created by pooling 10 breast milk samples with high OD values based on preliminary data. Ten samples from the final collection time (T2-3) of vaccinated mothers and COVID-19 positive mothers were selected for IgG and IgA, respectively. An arbitrary antibody concentration unit of 3000 was assigned to the highest OD value corresponding to the undiluted sample. The standard curve corresponds to eight 3-fold serial dilutions from 3000 to 1.38 arbitrary units. For samples at collection points T2-1 and T2-2 for IgA and IgG, respectively, undiluted and five 1:4 serial dilutions were used in order to determine the area under the curve (AUC) and endpoint titers. Endpoint titers were determined as the maximum dilution with an OD_450_ signal that is 2 standard deviations above the pre-pandemic control group and was calculated from log-transformed titration curves using a 4-parameter non-linear regression function in GraphPad Prism 8.4.3. Low endpoint titers that could not be modelized (*n*=3 for Oxford/AstraZeneca) were assigned a value of 0.1 for plotting and calculation purposes.

### Statistical analysis

**Fig. 1:**
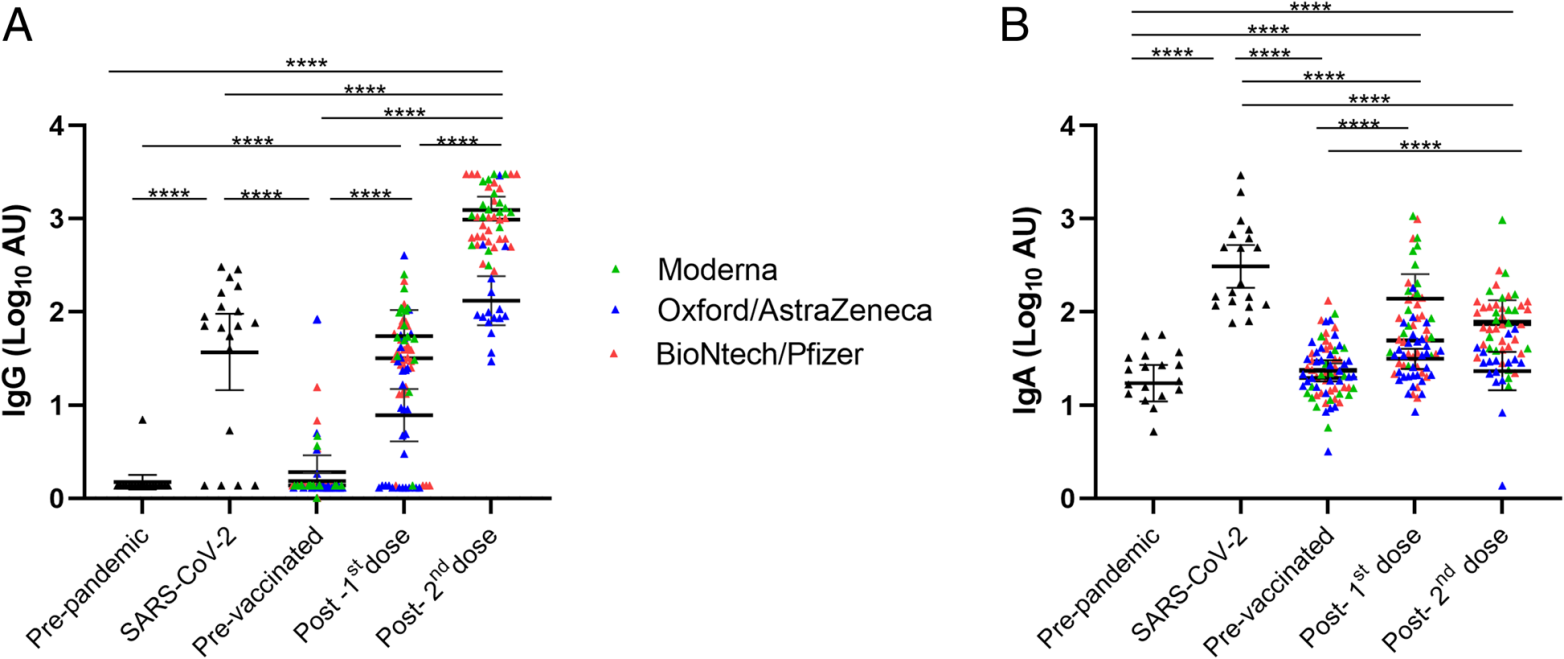
Effect of vaccination on IgG and IgA presence in breast milk. Results of IgG (**A**) and IgA (**B**) levels of the BioNtech/Pfizer, Moderna, and Oxford/AstraZeneca vaccines were grouped according to the post-vaccination days. Time points shown in the figure are as following: Post-1^st^ dose (2 weeks after 1^st^ dose of the three studied vaccines), post-2^nd^ dose (2 weeks after 2^nd^ dose). One-way ANOVA with a Tukey’s post hoc test for multiple comparisons was performed to assess the statistical significance between groups. Data is presented as mean and 95% of CI of the log-transformed arbitrary units (AU). Different colors represent the different vaccine types. All participants were included in the plots. *p <0.05, **p <0.01, ***p <0.001, and **** *p* <0.0001

GraphPad Prism 8.4.3 (GraphPad Software, San Diego, California, USA) was used for the statistical analysis. Antibody kinetics were fitted using a nonlinear 4-parameter least-square fit in GraphPad Prism 8.4.3. The resulted fit was used to model the tested samples calculating the arbitrary units of each sample. The maximum (3000 AU) or lowest (1.37 AU) values were assigned to those samples that could not be modelized because they were out of range in the OD values in the upper or the bottom part of the standard curve, respectively. To compare differences in AU, data were log-transformed. Mixed-effect analyses to accommodate missing values were performed to model longitudinal trends of anti-SARS-CoV-2 IgG and IgA. For the study of the general study of IgA and IgG levels (Fig. 1), Tukey’s test for multiple comparisons was used as a post hoc test in the mixed-effect analysis to compare IgA and IgG levels at 15 days after 1^st^ and 2^nd^ doses with the baseline condition. For the analysis of the percentage of positive samples, milk samples were classified as positive or negative considering the positive cut-off values for each Ig class calculated from the AU of the pre-pandemic control samples and defined as mean plus two standard deviations (SD). Chi-square test and Fisher’s exact test were used to analyze differences in the percentage of positive samples according to vaccines.

For trajectory studies, mixed-effects analyses were performed to model the complete course of vaccination for the three vaccines (Tukey’s multiple comparisons post hoc test). To perform the mixed-effects models in the three vaccines in the same model, data from the T1-3 and T2-0 were considered the same in the case of mRNA vaccines (since the vaccination course is shorter than in adenoviral-vectored vaccines). Kruskal-Wallis test with Dunn’s post hoc test for multiple comparisons was used to assess the differences in AUC and Endpoint titter values between the three vaccines. Other statistical evaluations were assessed using one-way ANOVA with Tukey’s post hoc test.

Chi-square test was used to assess potential associations between vaccine type and side effects. A *p*-value<0.05 was considered statistically significant.

## Results

### Study population characteristics

The study included eighty-six lactating women receiving COVID-19 vaccination (Table 1), with the age of 34.6 ± 3.7 years (mean±SD) and infant age of 12.7 ± 8.6 months (mean±SD). No significant differences in clinical variables were observed between the vaccine-type groups (Table 1). Mothers vaccinated with the adenoviral-vectored vaccine reported more side effects after vaccination compared to those vaccinated with the mRNA-based vaccines, including fever (*p*=0.0006) and headache (*p* <0.0001) (Additional file 1: Table S1). Seven infants developed a fever after maternal vaccination with no statistical differences between administered vaccine types. There were no serious adverse events during the study period.

### Anti-SARS-CoV-2 reactive antibodies in breast milk after vaccination

A strong reactivity was observed for IgG and IgA after vaccination mainly after the 2^nd^ dose (Fig. 1). Both IgG (Fig. 1A) and IgA (Fig. 1B) reached higher levels than those in the pre-pandemic group and those in the baseline time-point. The mixed-effects analysis also revealed that vaccination increased IgG (*p* <0.0001) and IgA (*p* <0.0001) levels in milk samples (Additional file 1: Table S2). While no differences were observed at baseline compared to pre-pandemic samples, 8 participants who reported SARS-CoV-2 infection prior to vaccination showed higher antibody levels compared to the baseline levels and were studied separately. Three other participants showed the same antibody profile but without confirmation of the previous infection. These 3 participants were not included in further analyses.

IgG levels after the 2^nd^ dose of the vaccines reached higher levels than those observed in the SARS-CoV-2 group (*p* <0.0001) (Fig. 1A), suggesting a strong response to the vaccines to generate IgG antibodies. Regarding IgA, low levels of non-specific binding were observed in both pre-pandemic and baseline milk samples (Fig. 1B). While anti-SARS-CoV-2 IgA levels significantly increased following the 1^st^ and the 2^nd^ dose (*p* <0.0001 and *p* <0.0001, respectively), they stayed lower than those found in the SARS-CoV-2 group (*p* <0.0001).

### Anti-SARS-CoV-2 antibody levels in milk according to vaccine type

**Fig. 2:**
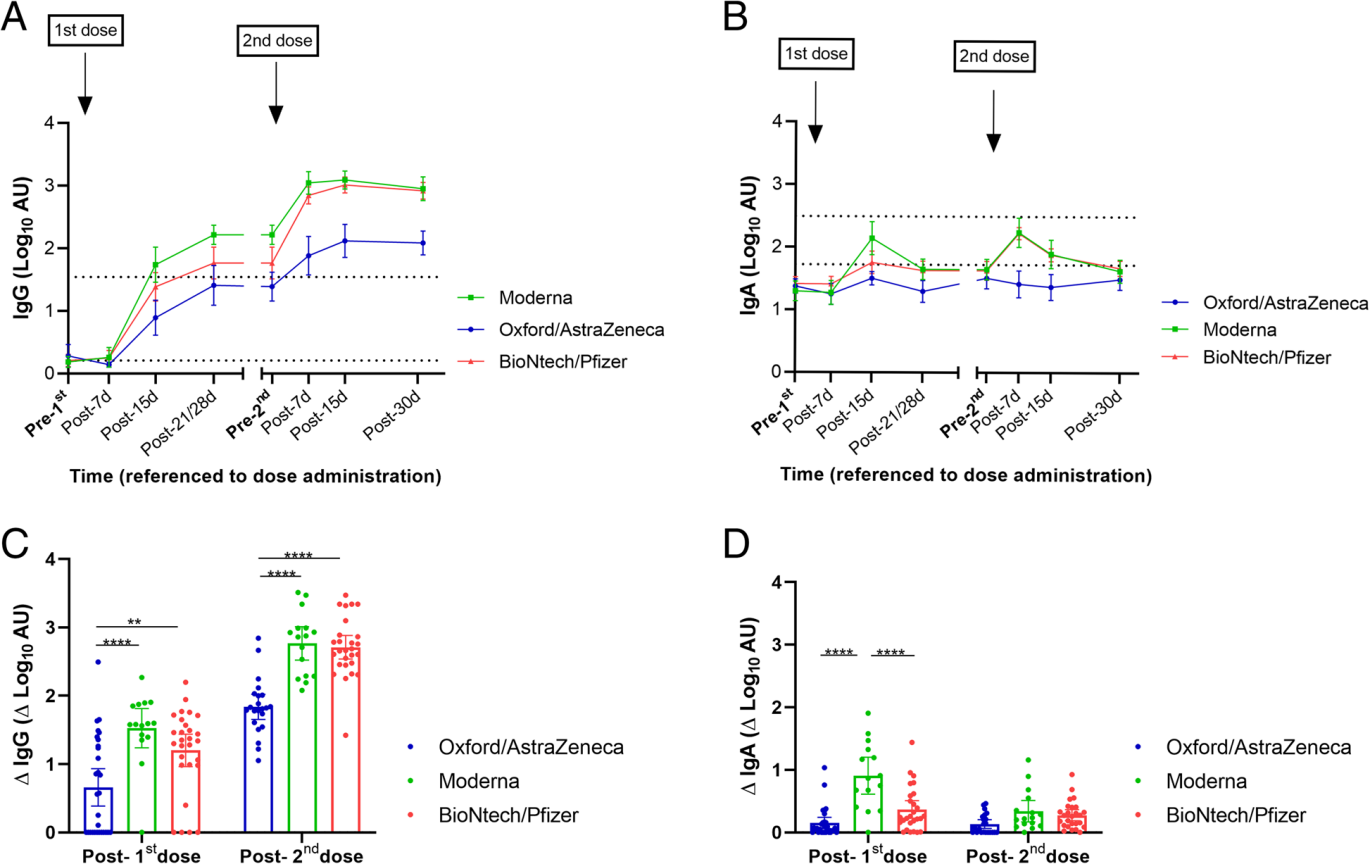
Effect of vaccination on IgG and IgA found in breast milk according to the administered vaccines in Spain. **A**, **B** Trajectories of the anti-SARS-CoV-2 IgG (**A**) and IgA (**B**) in breast milk samples according to vaccine type from baseline (before the 1^st^ dose) to 4 weeks post-vaccination course. The upper-dotted line in the plots of trajectories represent the mean of the log transformed arbitrary units (AU) for mothers with confirmed SARS-CoV-2 infection. The lower-dotted lines in the trajectories plot represent the established positive cut-off value (log of the mean+2SD of pre-pandemic anti-SARS-CoV-2 IgA and IgG AU). Mixed-effect analysis with multiple comparisons post hoc test was performed to test the significance in the antibody’s presence in breast milk and the differences according to vaccine type (Additional Table S4). *Y*-axis marked the approximated days of sampling. **C**, **D** Comparison of the increment in log-transformed AU from the determination of SARS-CoV-2 IgG (**C**) and IgA (**D**) from baseline to 2 weeks post 1^st^ dose and 30 days after 2^nd^ dose (at the end of the follow-up). One-way ANOVA with a Tukey’s post hoc test for multiple comparisons was performed to assess the statistical significance of the differences in the antibody detection. Data is presented as mean and 95% of CI of the log-transformed arbitrary units (AU). *p <0.05, **p <0.01, ***p <0.001, and **** *p* <0.0001

We next compared the immunogenic response induced by each vaccine type. While all three vaccines increased anti-SARS-CoV-2 IgG (Fig. 2A, Additional file 1: Figure S1) and IgA levels (Fig. 2B, Additional file 1: Figure S2), BioNtech/Pfizer and Moderna induced higher IgG levels than Oxford/AstraZeneca after administration of the 1^st^ dose. All three vaccine types induced their maximum effect 2 weeks after 2^nd^ dose (Fig. 2A, B) in terms of the IgG presence in breast milk. Furthermore, a higher percentage of samples from Moderna and BioNtech/Pfizer remained positive based on the established cut-off compared to Oxford/AstraZeneca 2 weeks after the 1^st^ dose (*p* <0.0001) (Additional file 1: Table S3). No major differences were found between the two mRNA-based vaccines, and all samples (from the three vaccines) were classified as positive after the 2^nd^ dose (Additional file 1: Table S3).

Mixed-effects analysis for the vaccination course revealed an effect of vaccine type on both IgG and IgA levels (*p* <0.0001), with higher immunoglobulin levels achieved after vaccination with mRNA-based Moderna and BioNTech/Pfizer compared to adenoviral-vectored Oxford/AstraZeneca vaccines at 2 and 3 weeks post-1^st^ dose (Additional file 1: Table S4).

**Fig. 3:**
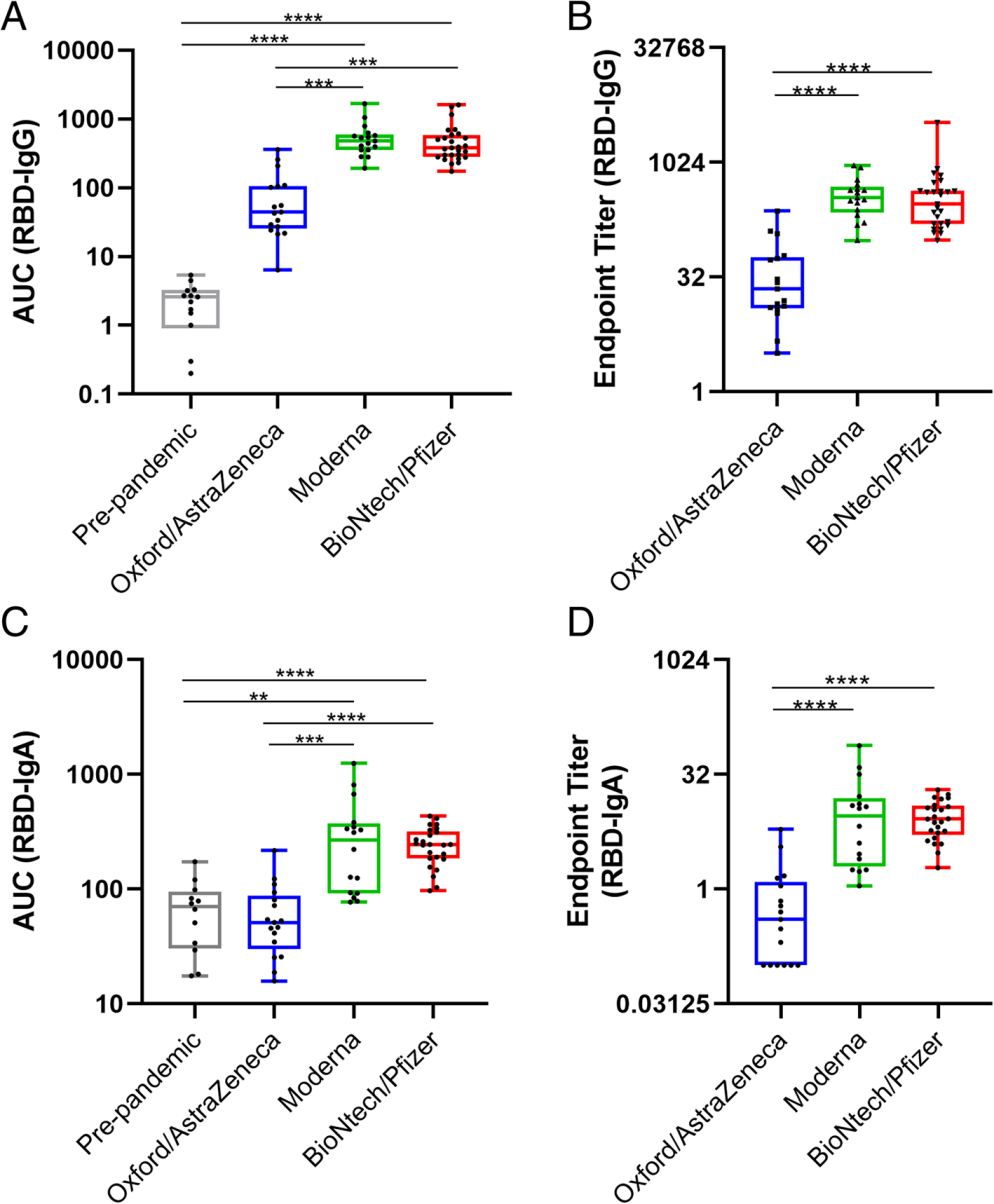
Effect of vaccine types on RBD-specific IgG and IgA endpoint titers and AUCs. AUCs (**A**, **C**) and endpoint titers (**B**, **D**) were calculated from titration curves for IgG (**A**, **B**) and IgA (**C**, **D**) at 15 days and 7 days post-2^nd^ dose, respectively. Endpoint titers were calculated for available samples from samples from Oxford/AZ (*n*=17), Moderna (*n*=16 and 17 for IgA and IgG, respectively), and BioNtech/Pfizer (*n*=25 and 27 for IgA and IgG, respectively) vaccinated mothers using the positive cut-off value established by pre-pandemic samples (*n*=13). Kruskal-Wallis test with a Dunn’s post hoc test for multiple comparisons was performed to assess the statistical significance of the differences in the antibody endpoint titer and AUC. Data is presented as individual points in box plots showing the median and max-min values. *p <0.05, **p <0.01, ***p <0.001, and ****p <0.0001

We then calculated the increase in IgG and IgA levels from baseline to 2 weeks after 1^st^ dose (for the three vaccines) and at the end of the follow-up period (30 days post-2^nd^ dose) (Fig. 2C, D). After both doses, Moderna and BioNtech/Pfizer vaccinated mothers showed a higher increment of milk anti-SARS-CoV-2 IgG compared to Oxford/AstraZeneca (*p* <0.0001), while anti-SARS-CoV-2 IgA levels were higher in both mRNA vaccines compared to Oxford/AstraZeneca after the 1^st^ dose but showed no differences at the end of the vaccination course according to vaccine type. After the 2^nd^ dose, no differences were observed between the two mRNA-based vaccines (Fig. 2C, D). Endpoint titer analysis and AUC calculations (Fig. 3) at the highest immunoglobulin detection (post-7 days for IgA and post-15 days for IgG after the 2^nd^ dose, respectively) also revealed higher antibody levels in breast milk samples from mRNA vaccines compared to those obtained from the adenoviral-vectored vaccine in both IgG (Fig. 3A, B) and IgA (Fig. 3C, D) with no differences between mRNA vaccines. Moreover, Oxford/AstraZeneca at 7 days after the 2^nd^ dose showed no significant IgA induction, with 70.6% of samples showing levels below the positive cut-off value based on the study of endpoint titer. In general, after the 2^nd^ dose, IgG levels reached higher levels compared to the 1^st^ dose while IgA levels did not further increase.

### Effects of vaccination in mothers with previous SARS-CoV-2 infection

**Fig. 4:**
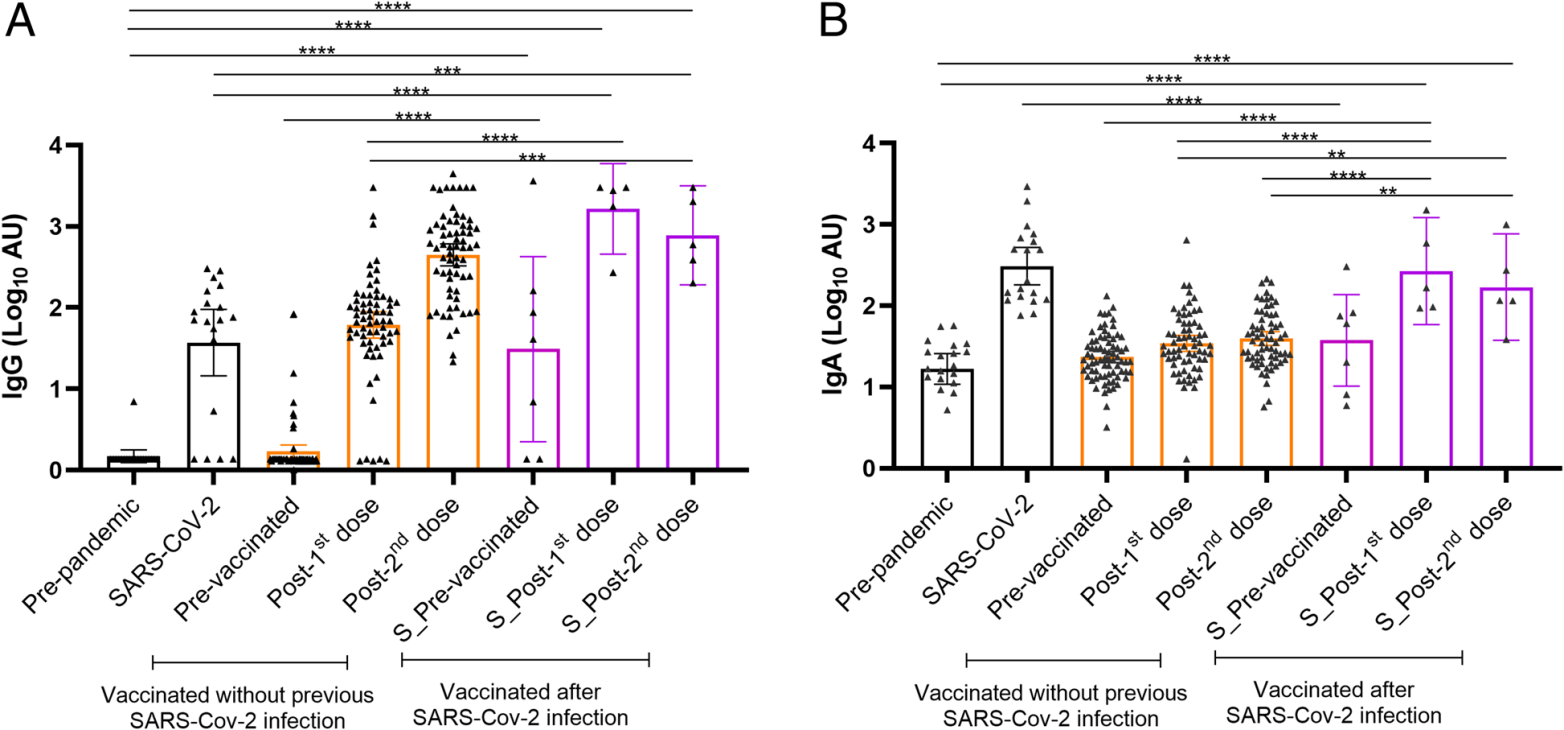
Effect of SARS-CoV-2 vaccination on antibody presence in breast milk in mothers with previous SARS-CoV-2 infection compared to those without previous exposure. Maternal samples from SARS-CoV-2-positive women (purple) before vaccination and those without viral exposure before vaccination (orange) were analyzed for detection of IgG (**A**) and IgA (**B**) antibody levels against RBD SARS-CoV-2. Additionally, samples from pre-pandemic and SARS-CoV-2-positive mothers were included. One-way ANOVA with a Tukey’s post hoc test for multiple comparisons was performed to assess the statistical significance of the differences in the antibody detection. Only those differences between the grouped samples of the vaccinated mothers with previous SARS-CoV-2 infection and all other analyzed groups are shown. The time points shown in the figure are as follows: Post-1^st^ dose and post-2^nd^ dose (21–28 days after 1^st^ dose and 2^nd^ dose, respectively). Data is presented as mean and 95% of CI of the log-transformed arbitrary units (AU). *p <0.05, **p <0.01, ***p <0.001, and **** *p* <0.0001

The subset of vaccinated mothers after SARS-CoV-2 infection (*n*=8) showed higher IgG and IgA levels at baseline (T1-0) when compared to the pre-pandemic group and participants without previous infection (Fig. 4). Pre-vaccination anti-SARS-CoV-2 IgG levels in milk were similar to those observed in the SARS-CoV-2 control group and higher than in the pre-pandemic group. IgG levels increased after the 1^st^ dose reaching values similar to those observed after the 2^nd^ dose in participants without previous exposure to SARS-CoV-2 (Fig. 4A). Baseline anti-SARS-CoV-2 IgA levels were lower compared to the positive control group (*p* <0.0001), reaching similar values after the 1^st^ dose of the vaccine (Fig. 4B).

## Discussion

This study reports the presence of anti-SARS-CoV-2 IgA and IgG in breast milk after vaccination, filling the gap in the knowledge about a potential vaccine-dependent immunogenic response. This study represents the biggest study up to now in Europe reporting specific antibody levels in milk for three different vaccines.

It has been reported that COVID-19 mRNA vaccination in pregnant and lactating women generated a robust serum immune response [12]. However, limited data are available on the impact of the different COVID-19 vaccines on breast milk. Breast milk acts as the main route of passive immunity from mother to infant after birth, which could be especially relevant in the pandemic context. Even though breast milk is considered the best nutrition source for infant development during the first months of life, there were uncertain guidelines for lactating women at the beginning of the pandemic. The presence of IgG and other specific antibodies against SARS-CoV-2 including IgA have been well studied after COVID-19 disease in serum and breast milk [19, 20]. However, information on their dynamics after vaccination remains limited. While accumulating data are being provided for mRNA-based vaccines [14, 21, 22], little information is available for the adenovirus-based vaccine (AstraZeneca) [23].

Our study shows increased levels of both anti-SARS-CoV-2 IgG and IgA levels after vaccination. The immunogenic response showed high intra- and inter-individual variability and was dependent on the vaccine type. Both IgA and IgG significantly increased at 2 weeks after the 1^st^ dose in all the analyzed vaccines and showed another peak after the 2^nd^ dose, except in the case of AstraZeneca for IgA, which showed no significant increase. While levels of anti-SARS-CoV-2 IgG were maintained at the final time point of the follow-up, in mRNA-based vaccines, IgA levels decreased 3–4 weeks after the 2^nd^ dose. These results are in accordance with previous studies focused on the presence of SARS-CoV-2 antibodies in breast milk [13, 22]. Indeed, we found that all samples remained positive for anti-SARS-CoV-2 IgG at the end of the vaccination course, while only 10–70% of the samples were classified as positive for IgA, which is in agreement with previously reported data [14]. Thus, our observations support vaccination as a useful clinical strategy to affect anti-SARS-CoV-2 IgG levels also in breast milk with the potential protective effect on the infant. Further studies with longer follow-up periods are needed for a deeper description of vaccination effects on antibody detection in breast milk as well as for the potential preventive effects in infants and to confirm the clinical relevance of the findings.

We observed a robust secretion of SARS-CoV-2 specific IgGs in breast milk after the 2^nd^ dose of vaccination, reaching higher levels than in naturally SARS-CoV-2-infected women, suggesting a powerful effect of vaccination on SARS-CoV-2 IgG production. In agreement with our data, a 2^nd^ mRNA vaccine dose increased the levels of anti-SARS-CoV-2 IgG but not IgA in both maternal blood and breast milk [12].

In mRNA-based vaccines, IgA secretion was evident as early as 2 weeks after vaccination followed by a peak after 4 weeks (a week after the 2^nd^ vaccine). Afterwards, levels of specific IgA against RBD gradually decrease during the 3–4 weeks post-vaccination. However, while anti-SARS-CoV-2 IgG levels were higher than those found in the SARS-CoV-2 group, IgA was lower compared to naturally infected women. Of note, this study did not address the presence of secretory antibodies, which are predominant in human milk as secretory IgA (sIgA). Secretory antibodies are expected to be more protective and resistant in mucosal environments. So far, only one study found low levels of secretory antibodies, highlighting the difference in antibody profile after intramuscular vaccination and natural infection [24].

Further studies with longer follow-ups are needed to track the persistence of IgA and sIgA specific antibodies. Indeed, we reported a higher baseline signal for anti-SARS-CoV-2 IgA than those found for IgG, which agrees with previous studies [16]. In this sense, a recent study reported higher S1 + S2-reactive IgA in breast milk from women that had viral respiratory infection symptoms before the pandemic than in milk from women without symptoms [25]. These data would partially explain the potential signal or cross-reactivity in breast milk samples before 2020 [25].

While different studies have reported the presence of specific IgA antibodies against SARS-CoV-2 in human milk after COVID-19 [3–5, 19], to our knowledge, none of the previous studies included the different vaccine types in the same analysis. While we observed no significant differences between the two mRNA-based vaccines, anti-SARS-CoV-2 IgG and particularly IgA levels in women receiving an adenoviral-vectored vaccine were lower compared to mRNA-based vaccines. This observation was also reported for IgG in serum samples [26]. In our study, we did not determine antibody titers in serum samples of lactating mothers. However, other authors showed a correlation between serum and milk antibody levels, with much lower titers in milk than in serum samples [22, 23, 27, 28]. Furthermore, two doses of the Oxford-AstraZeneca COVID-19 vaccine-induced lower levels of neutralizing antibodies against SARS-CoV-2 variants, including the Delta variant (B.1.617.2), than recipients of the Pfizer vaccine [29–31].

Indeed, we could not conclude the clinical relevance of this difference since it is well known that not only antibodies but also other immune components including memory and antigen-specific T cells could be activated by vaccines and play a role in the protection conferred by the adenoviral-vectored vaccines [32]. Nonetheless, our findings highlight that further studies with a bigger sample size and independent populations are needed in order to confirm our observations.

We also described the effect of vaccination in a small subset of mothers with a previous SARS-CoV-2 infection. Even though the small sample size, these mothers showed a high baseline IgG signal, similar to the positive control group and to that reported after the 1^st^ dose in mothers with no previous infection. Indeed, mothers with a previous SARS-CoV-2 infection reached the peak of anti-SARS-CoV-2 IgG levels after the 1^st^ dose, and these were comparable to those reached in the general studied population after the 2^nd^ dose of the vaccination course. Our results suggest that mothers with a previous infection could get a similar potential protective effect in their milk after a unique dose of the vaccination course. Recently, similar results in serum samples from health care workers have been reported [33], showing that specific IgG antibodies directed to different SARS-CoV-2 antigens (S1, S2, RBD, and N regions) persisted 3 weeks after a single vaccination [33]. Other studies, in agreement with our results, demonstrated that a single dose of mRNA-based vaccines elicited rapid immune responses in seropositive participants with the previous history of SARS-CoV-2 exposure [34, 35]. However, the persistence and duration of antibody responses in both milk and blood need further investigation. Nonetheless, further studies with a larger sample size focused on mothers with a history of SARS-CoV-2 infection could be crucial to confirm our results and to clarify the clinical relevance of these findings as well as the potential consequence for the vaccination programs.

### Limitations

Our study provides preliminary data, and it has limitations. Larger prospective studies, bigger samples sizes, and distinct cohorts in different locations are needed to confirm the safety of SARS-CoV-2 vaccination in breastfeeding women and the possible differences in the presence of antibodies in breast milk according to vaccine type. In addition, longer follow-up of the antibody levels would be needed in order to determine their persistence. Furthermore, although it has been suggested that antibodies found in breast milk would exert strong neutralizing effects, no functional assays were performed. In addition, the potential impact on neonatal growth as well as the potential protective effect against infection in the infant remains elusive.

## Conclusions

Breast milk from vaccinated women contains anti-SARS-CoV-2-specific IgA and IgG, with levels increasing considerably after a 2^nd^ dose in the case of IgG. Women with previous COVID-19 history increased antibody levels after the 1^st^ dose to the level observed in vaccinated women with no previous infection after the 2^nd^ dose. Further studies are needed to demonstrate the protective antibody effect against COVID-19 in infants from vaccinated and/or infected mothers and the differences observed by vaccine type.

## Supplementary Information

**Additional file 1: Table S1.** Reported maternal and infant side-effects after vaccination. **Table S2.** Results from the longitudinal mixed-effects analysis modeling the changes in IgG and IgA detection in human breast milk after vaccination. **Figure S1.** Individual trajectories of the SARS-CoV-2 IgG in breast milk samples according to vaccine from baseline (before the 1^st^ dose) to 3-4 weeks post vaccination course (A-C) and grouped by vaccine (D-F). Data is presented as log-transformed arbitrary units (AU) and AU ± 95% CI. **Figure S2.** Individual trajectories of the SARS-CoV-2 IgA in breast milk samples according to vaccine from baseline transformed arbitrary units (AU) and AU ± 95% CI. **Table S3.** Percentage of mothers with a signal above the established cut-off for a positive result for SARS-CoV-2 antibody presence according to vaccine. **Table S4.** Results from the longitudinal mixed-effects analysis modeling the changes in IgG and IgA detection in human breast milk at baseline and each analyzed time after vaccination according to vaccine.

**Additional file 2.** Raw data generated during the analysis of anti-SARS-Cov-2 IgG and IgA levels and used for statistical analysis.

## Data Availability

Information on the clinical characteristics of the participants included in the study is available in Table 1. The general reported maternal and infant side-effects after vaccination, and also, the details on individual trajectories of the SARS-CoV-2 IgG and IgA in breast milk samples are reported in Additional file 1. The raw data generated during the analysis of anti-SARS-Cov-2 IgG and IgA levels and used for statistical analysis is available in Additional file 2.
