# Supplementary material for: Anti-SARS-CoV-2 IgA and IgG in human milk after vaccination is dependent on vaccine type and previous SARS-CoV-2 exposure: a longitudinal study
Source: Genome Med. 2022 Apr 21;14:42. doi: 10.1186/s13073-022-01043-9 (PMC9022055; doi:10.1186/s13073-022-01043-9)
Supplement: Supplementary file 1 — Additional file 1: Table S1. Reported maternal and infant side-effects after vaccination. Table S2. Results from the longitudinal mixed-effects analysis modeling the changes in IgG and IgA detection in human breast milk after vaccination. Figure S1. Individual trajectories of the SARS-CoV-2 IgG in breast milk samples according to vaccine from baseline (before the 1st dose) to 3-4 weeks post vaccination course (A-C) and grouped by vaccine (D-F). Data is presented as log-transformed arbitrary units (AU) and AU ± 95% CI. Figure S2. Individual trajectories of the SARS-CoV-2 IgA in breast milk samples according to vaccine from baseline transformed arbitrary units (AU) and AU ± 95% CI. Table S3. Percentage of mothers with a signal above the established cut-off for a positive result for SARS-CoV-2 antibody presence according to vaccine. Table S4. Results from the longitudinal mixed-effects analysis modeling the changes in IgG and IgA detection in human breast milk at baseline and each analyzed time after vaccination according to vaccine. [file 13073_2022_1043_MOESM1_ESM.docx]

**Table S1.** Reported maternal and infant side-effects after vaccination.

|  | BioNtech/Pfizer | Moderna | Oxford/AstraZeneca | p-value |
| --- | --- | --- | --- | --- |
| Maternal side-effects | | | | |
| 1st dose | **N=34** | **N=20** | **N=32** |  |
| Local pain and tiredness | 31 (91.2%) | 18 (90%) | 28 (87.5%) | 0.885 |
| Fever | 3 (8.8%) | 0 (0·0%) | 12 (37.5%) | **0.0006** |
| Headache | 6 (17.6%) | 4 (20%) | 23 (71.9%) | **<0.0001** |
| Others (myalgia, insomnia, nausea) | 2 (5.8%) | 3 (15.0%) | 11 (34.4%) | **0.011** |
| 2nd dose | **N=32*** | **N=19*** | **N=29*** | **p-value** |
| Local pain and tiredness | 28 (87.5%) | 16 (84.2%) | 18 (62.1%) | **0.043** |
| Fever | 10 (31.2%) | 7 (35.0%) | 3 (10.3%) | 0.051 |
| Headache | 13 (40.6%) | 6 (30.0%) | 9 (31.0%) | 0.676 |
| Others (myalgia, insomnia, nausea) | 11 (34.4%) | 8 (40.0%) | 2 (6.9%) | **0.010** |
| Infant side-effects |  |  |  |  |
| 1st dose | **N=34** | **N=20** | **N=32** |  |
| Skin reaction (dermatitis, etc) | 0 (0·0%) | 1 (5.0%) | 2 (6.2%) | 0.352 |
| Fever | 1 (2.9%) | 0 (0·0%) | 0 (0·0%) | 0.461 |
| Irritability and insomnia | 2 (5.9%) | 0 (0·0%) | 5 (15.6%) | 0.111 |
| 2nd dose | **N=32*** | **N=19*** | **N=29*** | **p-value** |
| Skin reaction | 0 (0·0%) | 0 (0·0%) | 0 (0·0%) | - |
| Fever | 3 (9.4%) | 2 (10.5%) | 2 (6.9%) | 0.898 |
| Irritability and insomnia | 6 (17.6%) | 6 (31.5%) | 4 (13.8%) | 0.313 |

Data is expressed as positive cases (% of the total population). Chi-square test (three vaccines) and Exact-Fisher’s test (mRNA-based vaccines) were used to the comparisons for the side-effects between the analyzed vaccines. * Women with missing data from the second dose.

**Table S2.** Results from the longitudinal mixed-effects analysis modeling the changes in IgG and IgA detection in human breast milk after vaccination.

|  | Pre-vaccination | Post 1st dose | Post 2nd dose | Baseline vs 1st dose | p-value | Baseline vs  2^nd^ dose | p-value |
| --- | --- | --- | --- | --- | --- | --- | --- |
| SARS CoV-2 IgA | 1.37 ± 0.30 | 1.74 ±0.47 | 1.75 ± 0.42 | -0.37  (-0.50, -0.24) | <0.0001 | -0.38  (-0.50, -0.26) | <0.0001 |
| SARS CoV-2 IgG | 0.23 ± 0.34 | 1.27 ±0.72 | 2.80 ± 0.55 | -1.03  (-1.25, -0.82) | <0.0001 | -2.56  (-2.77, -2.35) | <0.0001 |

Data is presented as log-transformed arbitrary units. The following time points were considered in the model: post-1st dose (2 weeks after 1^st^ dose of the three studied vaccines), post- 2nd dose (3-4 weeks after 2^nd^ dose of the mRNA-based vaccines). The first three columns present the mean of log-transformed arbitrary units ± SD and the comparisons columns present the mean difference (95% confidence interval).


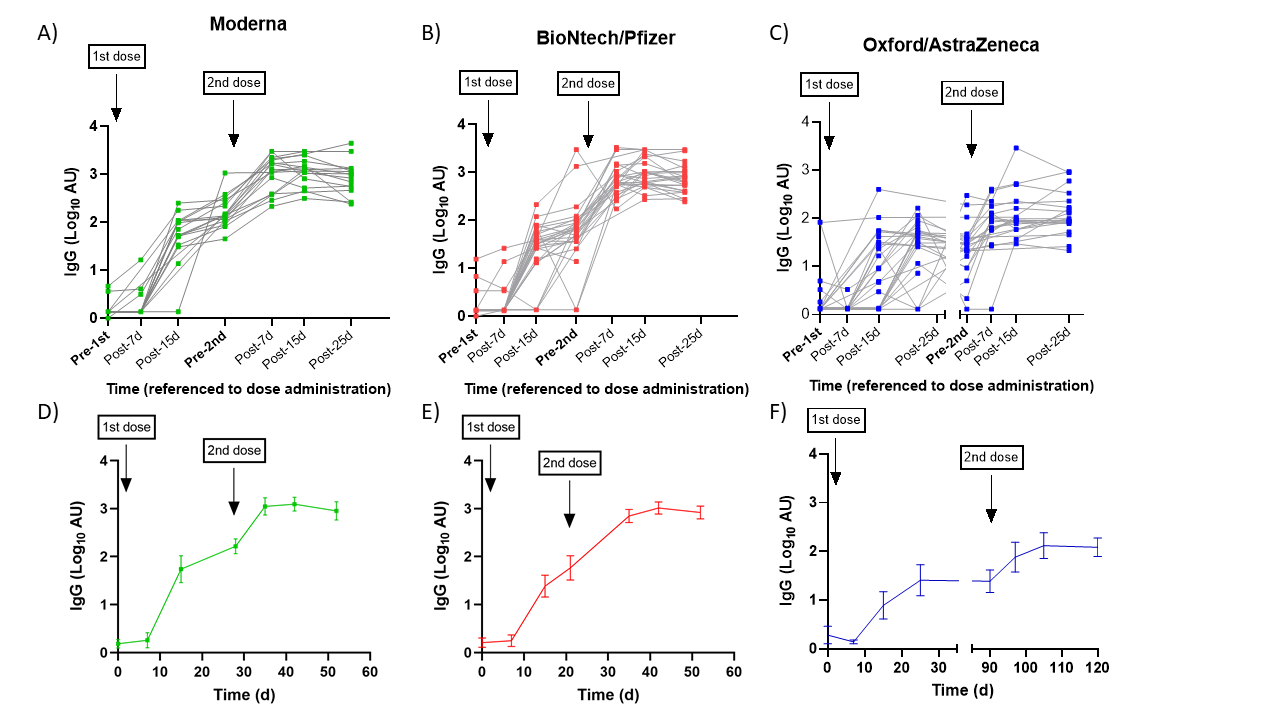


**Figure S1.** Individual trajectories of the SARS-CoV-2 IgG in breast milk samples according to vaccine from baseline (before the 1^st^ dose) to 3-4 weeks post vaccination course (A-C) and grouped by vaccine (D-F). Data is presented as log-transformed arbitrary units (AU) and AU ± 95% CI.


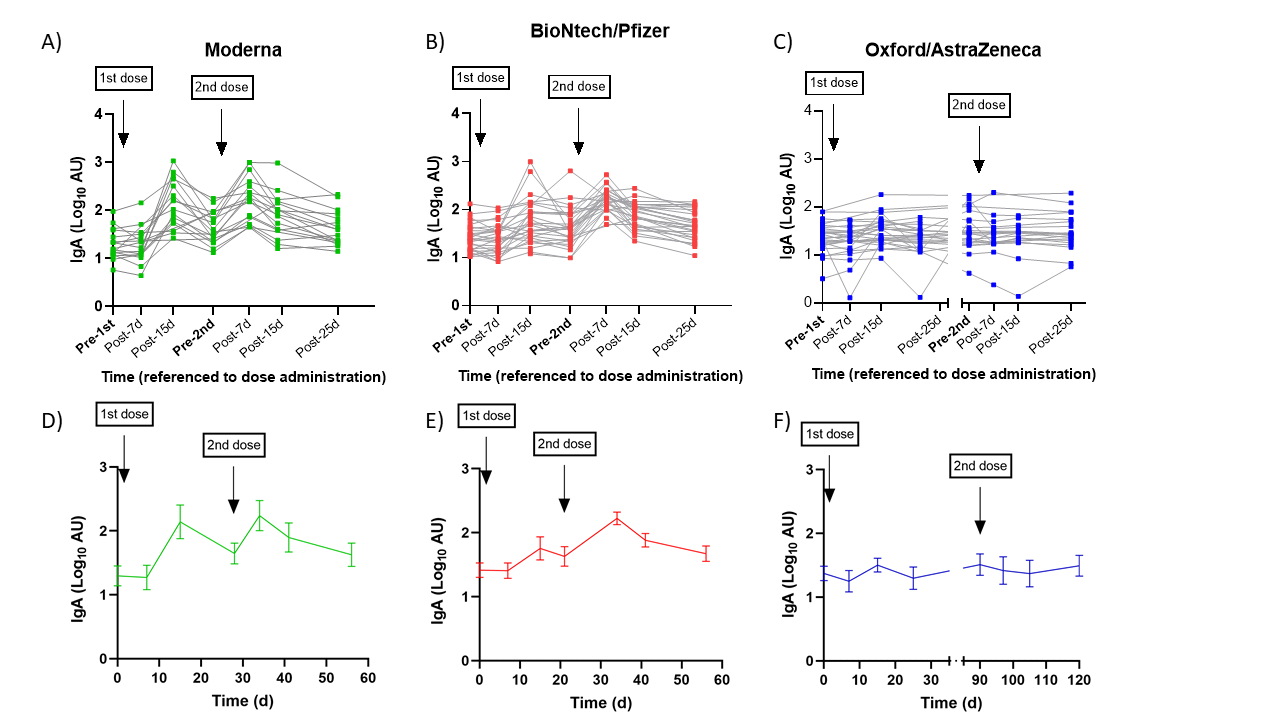


**Figure S2.** Individual trajectories of the SARS-CoV-2 IgA in breast milk samples according to vaccine from baseline transformed arbitrary units (AU) and AU ± 95% CI.

**Table S3.** Percentage of mothers with a signal above the stablished cut-off for a positive result for SARS-CoV-2 antibody presence according to vaccine.

|  | Moderna  (n=18) | BioNtech  Pfizer  (n=28) | Oxford  AstraZeneca  (n=29) | p-value |
| --- | --- | --- | --- | --- |
| IgG |  |  |  |  |
| 2 weeks  Post 1st dose | 93.8% | 85.7% | 62.1% | **<0.0001** |
| 3 weeks  Post 1st dose | 100% | 92.9% | 84.2% | **0.0001** |
| 2 weeks  Post 2nd dose | 100% | 100% | 100% | >0.999 |
| 3-4 weeks  Post 2nd dose | 100% | 100% | 100% | >0.999 |
| IgA |  |  |  |  |
| 2 weeks  Post 1st dose | 81.3% | 46.4% | 17.2% | **<0.0001** |
| 3 weeks  Post 1st dose | 50% | 32.1% | 5.3% | **<0.0001** |
| 2 weeks  Post 2nd dose | 64.7% | 66.7% | 12.5% | **<0.0001** |
| 3-4 weeks  Post 2nd dose | 35.3% | 40.7% | 27.3% | 0.112 |

The positive cut-off was stablished as a signal above the mean + 2 SD of the arbitrary units from the prepandemic group. Samples with previous SARS-CoV-2 infection (n=8) and those with a similar antibody profile (n=3) were removed from the analysis. Chi-square test were used to test significant differences in the number of positive samples according to vaccines. There are some missing data from some of the participants.

**Table S4.** Results from the longitudinal mixed-effects analysis modeling the changes in IgG and IgA detection in human breast milk at baseline and each analyzed time after vaccination according to vaccine.

|  | Moderna | BioNtech  /Pfizer | Oxford  AstraZeneca | Moderna vs BioNtech/Pfizer | Moderna vs Oxford | Oxford vs BioNtech/Pfizer |
| --- | --- | --- | --- | --- | --- | --- |
| SARS CoV-2 IgG | | | | | | |
| T1-0 | 0.186 ± 0.17 | 0.208 ± 0.25 | 0.283 ± 0.47 | -0.023  (-0.172, 0.127)  0.929 | 0.097  (-0.138, 0.332)  0.578 | 0.074  (-0.167, 0.315)  0.735 |
| T1-1 | 0.257 ± 0.29 | 0.247 ± 0.31 | 0.140 ± 0.09 | 0.010  (-0.222, 0.241)  0.994 | -0.012  (-0.312, 0.078)  0.300 | -0.107  (-0.261, 0.046)  0.214 |
| T1-2 | 1.738 ± 0.53 | 1.384 ± 0.59 | 0.893 ± 0.74 | 0.354  (-0.068, 0.776)  0.115 | -0.845  (-1.308, -0.382)  **0.0002** | -0.491  (-0.917, -0.065)  **0.020** |
| T1-3 | 2.215 ± 0.31 | 1.767 ± 0.66 | 1.409 ± 0.66 | 0.448  (0.099, 0.798)  **0.009** | 0.878  (0.473, 1.283)  **0.0002** | -0.422  (-0.932, 0.089)  0.172 |
| T2-0 | 2.215 ± 0.31 | 1.767 ± 0.66 | 1.388 ±0.53 | 0.448  (0.099, 0.798)  **0.009** | -0.827  (-1.152, -0.503)  **<0.0001** | -0.379  (-0.781, 0.023)  0.069 |
| T2-1 | 3.045 ± 0.35 | 2.844 ± 0.33 | 1.881 ± 0.58 | 0.201  (-0.065, 0.466)  0.167 | -1.165  (-1.584, -0.745)  **<0.0001** | -0.964  (-1.364, -0.563)  **<0.0001** |
| T2-2 | 3.091 ± 0.28 | 3.011 ± 0.32 | 2.119 ±0.49 | 0.081  (-0.143, 0.305)  0.656 | -0.972  (-1.325, -0.619)  <0.0001 | -0.891  (-1.237, -0.545)  <0.0001 |
| T2-3 | 2.95 ± 0.37 | 2.918 ± 0.33 | 2.085 ± 0.43 | 0.032  (-0.240, 0.305)  0.954 | -0.866  (-1.179, -0.553)  **<0.0001** | -0.833  (-1.107, -0.560)  **<0.0001** |
| SARS CoV-2 IgA | | | | | | |
| T1-0 | 1.296 ± 0.31 | 1.413 ± 0.29 | 1.372 ± 0.30 | -0.118  (-0.344, 0.108)  0.418 | 0.076  (-0.151, 0.303)  0.692 | -0.042  (-0.230, 0.147)  0.856 |
| T1-1 | 1.270 ± 0.36 | 1.407 ± 0.31 | 1.249 ± 0.37 | -0.137  (-0.403, 0.129)  0.422 | -0.021  (-0.317, 0.275)  0.984 | -0.158  (-0.401, 0.085)  0.265 |
| T1-2 | 2.142 ± 0.49 | 1.754 ±0.47 | 1.500 ± 0.29 | 0.388  (0.015, 0.761)  **0.040** | -0.642  (-0.980, -0.304)  **0.0003** | -0.254  (-0.503, -0.005)  **0.044** |
| T1-3 | 1.647 ± 0.33 | 1.630 ± 39 | 1.296 ± 0.36 | 0.017  (-0.244, 0.279)  0.986 | -0.351  (-0.630, -0.072)  **0.011** | -0.334  (-0.606, -0.063)  **0.013** |
| T2-0 | 1.647 ± 0.33 | 1.630 ± 0.39 | 1.509 ±0.39 | 0.017  (-0.244, 0.279)  0.986 | -0.138  (-0.411, 0.135)  0.442 | -0.121  (-0.386, 0.145)  0.518 |
| T2-1 | 2.239 ± 0.46 | 2.224 ± 0.24 | 1.416 ± 0.40 | 0.016  (-0.287, 0.317)  0.991 | -0.823  (-1.191, -0.455)  <0.0001 | -0.808  (-1.087, -0.529)  <0.0001 |
| T2-2 | 1.896 ± 0.44 | 1.880 ± 0.27 | 1.369 ± 0.39 | 0.016  (-0.282, 0.314)  0.991 | -0.527  (-0.884, -0.170)  **0.003** | -0.512  (-0.787, -0.236)  **0.0003** |
| T2-3 | 1.625 ± 0.36 | 1.672 ± 0.30 | 1.491 ± 0.37 | -0.046  (-0.303, 0.210)  0.897 | -0.135  (-0.419, 0.150)  0.487 | -0.181  (-0.419, 0.057)  0.167 |

Data is presented as log-transformed arbitrary units. Results in the comparison columns are shown as mean difference (95% confidence interval) and p-value. Significant p-values are marked in bold. To perform the mixed-effects models in the three vaccines in the same model, data from the T1-3 and T2-0 were considered the same in the case of mRNA vaccines. Time points are expressed as follows: pre-vaccination (T1-0: 0 days), 1 week (T1-1), 2 weeks (T1-2) and 3 weeks (T1-3) post the 1^st^ dose of vaccine; and a one day before 2^nd^ dose (T2-0), 1 week (T2-1), 2 weeks (T2-2) and 3-4 weeks (T2-3) post 2^nd^ dose of vaccine.
